# Keeping translational research grounded in human biology

**DOI:** 10.1172/JCI178332

**Published:** 2024-01-16

**Authors:** Saptarsi M. Haldar

**Affiliations:** 1 Amgen Research, South San Francisco, California, USA.; 2 UCSF, San Francisco, California, USA.; 3 Gladstone Institutes, San Francisco, California, USA.

Developing new and effective medicines is perhaps one of the most important, yet difficult endeavors that we pursue as a society. As a drug developer, I am part of a team wrestling with a daunting task — picking the most promising drug targets from the myriad biological pathways in the human body, designing drug molecules that can be administered to patients to modify these targets in a manner that impacts disease with an acceptable safety profile, and demonstrating the efficacy and value of these medicines in clinical trials. These hurdles contribute to the extremely low success rate of drug development programs progressing from target identification to approval (1). Increasingly, drug developers recognize that the most important consideration for improving the chance of success is to integrate data on human diversity (genomics, transcriptomics, proteomics, and other forms of molecular and phenotypic data) (2), from target nomination through all subsequent stages of drug development.

## Using human biological insights to inform drug development

The main reason investigational programs fail in clinical testing is lack of efficacy (3), often because the therapeutic hypothesis was flawed from the outset. In many cases, the target was never a part of a biochemical pathway that is perturbed in the human disease, despite provocative correlations or therapeutic effects in cell and animal models. Even when the target induces a specific mechanistic biological effect, the target biology may differ in humans due to differences in the primary pathway or alternative pathways that circumvent the therapeutic intervention. In cases where research teams are “on the right track” with a sound therapeutic hypothesis and drug candidate that meets key technical criteria, clinical trial participants are genetically and phenotypically diverse, with different environmental exposures, making any potentially observable biological effect prone to substantial variability, with reduced efficacy in some individuals. Given these challenges, we must embark on the drug development journey with the highest possible conviction in 3 things: (a) that a specific target, pathway, or biological process is relevant in the human disease of interest; (b) that we can quantify target engagement by the drug in a manner that relates to the target’s mechanism of action; and (c) that we can identify a patient population in which the therapeutic intervention can demonstrate meaningful clinical benefit. Data on human diversity can be invaluable in informing each of these steps.

Genomes continuously evolve through random variation and selection, leading to the enormous genetic and phenotypic diversity observed in humans. This evolution has been nonlinear and nonuniform, being influenced by selective population growth. Our evolutionary history thus provides a rich resource of human genome variation that can be mined and harnessed for drug discovery. Information from whole-genome sequencing, transcriptomics, proteomics, structural prediction modeling, and detailed phenotypic assessments (including electronic health records), coupled with both hypothesis-free and hypothesis-informed analytical methods, can be utilized to identify pathways linked to phenotypes of interest. This knowledge forms a powerful foundation for drug developers to prioritize targets that are more likely to be safe and effective when pharmacologically interdicted in humans. Information on patient heterogeneity can also help clinical trialists to better identify patient subsets that may preferentially respond to a specific therapeutic intervention.

## Targeting PCSK9: a model for integrating human genetics

Drug development for atherosclerotic cardiovascular disease (ASCVD) serves as a powerful example for how pursuing drug development in the context of human biological diversity can improve success rates, and highlights principles that can be applied to all human disease. Despite established interventions that include the statin class of low-density lipoprotein cholesterol–lowering (LDL-C–lowering) agents, incidence of ASCVD and its sequelae (e.g., death, myocardial infarction, heart failure, stroke) continues to increase worldwide (4), representing a substantial societal burden with an unmet need for new therapies. However, addressing this need in an already-crowded pharmacological landscape requires identification of novel and effective pathways, as well as drug mechanisms that are plausible and scalable. First, a new drug must meaningfully improve patient outcomes (e.g., decrease heart attacks) when added on top of established standard of care in a randomized trial of highly heterogeneous patients. Second, the most effective ASCVD therapies are preventive and taken chronically, making the bar for safety very high, even for high-risk patients. Third, while a therapeutic intervention may elicit an intended physiological effect in humans (e.g., altering a plasma lipid species, decreasing platelet activation, improving endothelial function, decreasing an inflammatory mediator) in early-phase clinical studies, these short-term changes do not necessarily indicate that clinical outcomes will be improved. Moreover, preclinical models of atherosclerosis are principally driven by hypercholesterolemia, are short-term perturbations (weeks to months) compared with the protracted course of human ASCVD (decades), mainly assess arterial plaque burden, and fail to recapitulate the event-driven nature of human disease progression. Given these obstacles, it is simply too risky to pursue drug targets in ASCVD in the contemporary era without being grounded in molecular insights into the relevant aspects of human diversity.

The development of proprotein convertase subtilisin/kexin type 9 (PCSK9) inhibitors is perhaps one of the finest examples of how human genetics can catalyze translational research, providing guiding principles for future cardiovascular drug development. In 2003, rare coding variants in *PCSK9*, which was then an obscure gene, were reported to cause hypercholesterolemia in humans (5). Subsequently, the Dallas Heart Study investigators identified presumed loss-of-function variants in *PCSK9* that were associated with lower LDL-C and lower incidence of cardiovascular events (6). While these human genetic observations were the basis for pursuing PCSK9 inhibition for LDL-C lowering and cardiovascular prevention, preclinical studies that were pursued in the context of the human genetics were also part of the landscape for drug discovery. Basic mechanistic studies in cultured cells and mouse models indicated that PCSK9 underwent rapid autoproteolysis into a mature form that triggered endosomal trafficking and intracellular degradation of the LDL receptor, culminating in decreased cell surface receptor density and increased plasma LDL-C concentration (7–11). Structure-function analyses of PCSK9 confirmed that the protective variants were indeed loss-of-function variants with defective synthesis, trafficking, or secretion, while variants associated with hypercholesterolemia featured gain-of-function mechanisms that increased interactions between PCSK9 and LDL receptors (7, 12).

These preclinical studies and others, when interpreted in the context of compelling human genetics, impacted translation. First, the genetic and preclinical studies together confirmed that PCSK9 loss of function was protective, supporting inhibition as the therapeutic approach — it is much easier to engineer a drug molecule to potently inhibit a target than to activate it. In addition, the identification of protective loss-of-function variants suggested that inhibiting this target may be generally relevant to myocardial infarction risk and not just to the individuals with gain-of-function mutations. Second, biochemical studies suggested that PCSK9 did not have proteolytic activity against other proteins involved in LDL-C homeostasis such as the LDL receptor, but rather drove rapid intracellular autoproteolysis to produce a mature form of PCSK9. To date, efforts to directly inhibit PCSK9 proteolytic activity through small molecules have been unsuccessful. Other potential druggable steps included the cellular synthesis of PCSK9 and the enigmatic interaction of PCSK9 with the LDL receptor. Eventually, a pioneering class of therapeutic antibodies that neutralized the PCSK9–LDL receptor interaction and reduced plasma LDL-C was successfully developed (13). That work was supported by mapping and detailed structural resolution of the interface between PCSK9 and the LDL receptor, which explained how those antibodies achieve neutralization (see international patent publication WO 2009/026558; ref. 14).

In addition to catalyzing therapeutic molecule discovery, human genetic diversity analyses helped de-risk potential safety concerns with PCSK9 inhibition and provided a degree of conviction in clinical efficacy that was necessary to justify pursuit of large cardiovascular outcomes studies, such as the testing of evolocumab in the FOURIER trial (15). The human variants confirmed that loss of function of only a single gene was sufficient to confer substantial LDL-C lowering and cardiovascular protection. Subsequent human genetic analyses provided convincing support that the cardioprotective effect of PCSK9 inhibition was through LDL-C lowering (16) and thereby helped researchers estimate the risk reduction that might occur when PCSK9 inhibitors were added to baseline statin therapy. Importantly, human genetic analyses have not supported the hypothesis that raising the plasma concentration of high-density lipoprotein (HDL) particles would reduce atherosclerotic cardiovascular events (17), indicating the need to focus on therapies to lower plasma LDL-C and other causal non-HDL cholesterol species. An antibody against murine PCSK9 developed in the course of the evolocumab program elicited a 26% to 36% reduction in total cholesterol in adult mice (though unlike primates, mice carry the majority of their circulating cholesterol in HDL particles) (13). These results, along with the human genetics, provided the critical impetus to press forward with nonhuman primate studies (where 80% LDL reduction was observed) (13) and subsequent first-in-human testing of evolocumab. Finally, human genetic analyses supported the long-term safety of PCSK9 inhibition (and the low levels of plasma LDL-C that would be achieved), mitigating concerns of adverse neurocognitive effects, incident diabetes, and other potential liabilities (18), concerns which have been further alleviated with data from large randomized controlled trials and long-term use of approved PCSK9 inhibitors (19).

## Applying lessons learned from PCSK9 inhibitor development

In retrospect, PCSK9 is an atypical case where the genetic findings provided a compelling path to target validation and made a case for translatability. Most genetic associations will have smaller effect sizes or more obscure targets, requiring more effort for determining translatability. However, the insights gleaned from PCSK9 illustrate the power of this approach and inform how population-scale interrogation of human biological diversity can impact drug development for common diseases.

While aggressive LDL-C lowering is a cornerstone of cardiovascular prevention, many patients with low plasma LDL-C still have cardiovascular events, a term called “residual cardiovascular risk.” Genetic studies have identified several new drug targets that may confer cardiovascular protection in an LDL-C–independent manner (20). One of the most interesting targets is the circulating lipoprotein “little a” particle [Lp(a)] (21), which is encoded by the *APOA* gene and produced by the liver. Lp(a) can be inhibited with small interfering RNA or antisense oligonucleotide drugs that are directed against *APOA* in hepatocytes, approaches that are being tested in ongoing phase III outcome studies (22). As orthologs for human Lp(a) are only found in old-world primates, human genetic studies are essential to elucidate any putative pathogenic mechanism and develop drugs to block potential pathological effects. Indeed, human genetic and proteomic analyses may help define optimal plasma Lp(a) cutoffs and other enrichment factors for trial inclusion, select the most appropriate clinical trial endpoints, quantify any dose-response relationship between Lp(a) lowering and reduction in cardiovascular events, de-risk the potential liabilities of very low plasma Lp(a) concentration, and understand the implications of higher mean plasma Lp(a) concentration associated with African ancestry.

In addition to helping identify the best drug targets, human genetics and proteomics have enormous potential to enhance the design and conduct of clinical trials. As the standard of care for preventing atherosclerotic events continues to improve, demonstrating large effects on absolute risk reduction in a broad population using standard clinical criteria for trial enrollment will be increasingly difficult. While a single common sequence variant is typically associated with only a modest incremental increase in the risk of disease, genetic risk scores based on the aggregate effect of many common variants can be predictors of incident cardiovascular events that may provide discriminatory information beyond traditional clinical risk factors in certain populations (23, 24). Therefore, genetic risk scores may be used to enrich clinical trials for participants who are more likely to have cardiovascular events during the trial timeframe, thereby magnifying the demonstrable efficacy of a drug and potentially allowing trials to be smaller, faster, and cheaper (25). In some cases, genetic risk scores may also be able to enrich a trial with patients who preferentially benefit from a specific intervention (26).

## Beyond genetics

A limitation of genetic risk scores is that the germline genome sequence is static. By contrast, human disease pathogenesis involves dynamic interaction between the environment and the output of the genome (e.g., RNA and proteins). Furthermore, while the germline genome may help assess the lifetime risk of a disease, it may not robustly influence disease progression or predict response to therapy. By comparison, the plasma proteome, which is under the influence of genetic variation, changes in response to environmental factors such as stress, injury, aging, food intake, disease progression, and drug exposure. The plasma proteome may therefore help capture the biology of gene-environment interactions. High-throughput proteomic technologies that allow for simultaneous measurement of thousands of proteins in the plasma using affinity-based methods are a powerful complement to genome sequencing. When applied at population scale, proteomics can provide information regarding how sequence variants impact human traits and diseases, identify plasma proteins that can serve as biomarkers for target engagement or patient selection, and in some cases identify drug targets that would not necessarily emerge from germline genetic analyses alone. Applying artificial intelligence–based analytical methods may enhance the ability to extract insights from these vast amounts of genetic, transcriptomic, proteomic, and clinical data. For example, deCODE Genetics has recently developed an artificial intelligence model based on plasma proteomics to predict what is left of life (27) and has also validated a proteomic risk score for prediction of atherosclerotic cardiovascular events that has superior performance to genetic risk scores (28). Given the dynamic nature of the proteome, such proteomic risk scores could change with drug treatment, potentially providing an early surrogate for cardiovascular events prior to the completion of a large, protracted, and expensive outcome study. These proteomics-based patient enrichment strategies may also enhance our ability to demonstrate outsized efficacy in clinical trials and observe meaningful treatment effects at earlier time points.

## Conclusions

I hope that my perspective as a scientist who is deeply engaged in discovering and developing medicines, framed in the context of atherosclerosis, highlights the importance of staying grounded in human biology when pursuing disease-related research. While signals emanating from human diversity data will not always be as compelling as those seen with PCSK9 or Lp(a), I believe these examples provide key lessons that are broadly applicable to many human diseases and can help improve our chances of translating scientific discoveries into impactful new medicines.

## Version 1 01/16/2024

Electronic publication
